# Use of Massage Therapy for Pain, 2018-2023: A Systematic Review

**DOI:** 10.1001/jamanetworkopen.2024.22259

**Published:** 2024-07-15

**Authors:** Selene Mak, Jennifer Allen, Meron Begashaw, Isomi Miake-Lye, Jessica Beroes-Severin, Gerardo De Vries, Emily Lawson, Paul G. Shekelle

**Affiliations:** 1 Veterans Health Administration, Greater Los Angeles Healthcare System, Los Angeles, California; 2 UCLA Fielding School of Public Health, University of California, Los Angeles; 3 RAND Corporation, Santa Monica, California

## Abstract

**Importance:**

Massage therapy is a popular treatment that has been advocated for dozens of painful adult health conditions and has a large evidence base.

**Objective:**

To map systematic reviews, conclusions, and certainty or quality of evidence for outcomes of massage therapy for painful adult health conditions.

**Evidence Review:**

In this systematic review, a computerized search was conducted of PubMed, the Allied and Complementary Medicine Database, the Cumulated Index to Nursing and Allied Health Literature, the Cochrane Database of Systematic Reviews, and Web of Science from 2018 to 2023. Included studies were systematic reviews of massage therapy for pain in adult health conditions that formally rated the certainty, quality, or strength of evidence for conclusions. Studies of sports massage therapy, osteopathy, dry cupping or dry needling, and internal massage therapy (eg, for pelvic floor pain) were ineligible, as were self-administered massage therapy techniques, such as foam rolling. Reviews were categorized as those with at least 1 conclusion rated as high-certainty evidence, at least 1 conclusion rated as moderate-certainty evidence, and all conclusions rated as low- or very low–certainty evidence; a full list of conclusions and certainty of evidence was collected.

**Findings:**

A total of 129 systematic reviews of massage therapy for painful adult health conditions were found; of these, 41 reviews used a formal method to rate certainty or quality of evidence of their conclusions and 17 reviews were mapped, covering 13 health conditions. Across these reviews, no conclusions were rated as high certainty of evidence. There were 7 conclusions that were rated as moderate-certainty evidence; all remaining conclusions were rated as low- or very low–certainty evidence. All conclusions rated as moderate certainty were that massage therapy had a beneficial associations with pain.

**Conclusions and Relevance:**

This study found that despite a large number of randomized clinical trials, systematic reviews of massage therapy for painful adult health conditions rated a minority of conclusions as moderate-certainty evidence and that conclusions with moderate- or high-certainty evidence that massage therapy was superior to other active therapies were rare.

## Key Points

**Question:**

What is the certainty or quality of evidence in recent systematic reviews for use of massage therapy for painful adult health conditions?

**Findings:**

This systematic review identified 129 systematic reviews in a search of the literature published since 2018; of these, 41 assessed the certainty or quality of evidence of their conclusions. Overall, 17 systematic reviews regarding 13 health conditions were mapped, and most reviews concluded that the certainty of evidence was low or very low.

**Meaning:**

This study found that despite massage therapy having been the subject of hundreds of randomized clinical trials and dozens of systematic reviews about adult health conditions since 2018, there were few conclusions that had greater than low certainty of evidence.

## Teaser

This systematic review maps the certainty and quality of evidence reported by systematic reviews in 2018 to 2023 of massage therapy for pain in adults.

## Introduction

Massage therapy is a popular and widely accepted complementary and integrative health modality for individuals seeking relief from pain.^1^ This therapy is the practice of manual assessment and manipulation of the superficial soft tissues of skin, muscle, tendon, ligament, and fascia and the structures that lie within the superficial tissues for therapeutic purpose.^2^ Individuals may seek massage therapy to address pain where conventional treatments may not always provide complete relief or may come with potential adverse effects. Massage therapy encompasses a range of techniques, styles, and durations and is intended to be delivered by uniquely trained and credentialed therapists.^3^ Original research studies have reported on massage therapy delivered by a wide variety of health care professionals, such as physical therapists, physiotherapists, and nurses.^4,5^ Despite massage therapy’s popularity and long history in practice, evidence of beneficial outcomes associated with massage therapy remains limited.

The Department of Veterans Affairs (VA) previously produced an evidence map of massage therapy for pain, which included systematic reviews published through 2018.^6^ An evidence map is a form of systemic review that assesses a broad field to identify the state of the evidence, gaps in knowledge, and future research needs and that presents results in a user-friendly format, often a visual figure or graph.^7^ To categorize this evidence base for use in decision-making by policymakers and practitioners, VA policymakers requested a new evidence map of reviews published since 2018 to answer the question “What is the certainty of evidence in systematic reviews of massage therapy for pain?”

## Methods

This systematic review is an extension of a study commissioned by the VA. While not a full systematic review, this study nevertheless reports methods and results using the Preferred Reporting Items for Systematic Reviews and Meta-analyses (PRISMA) reporting guideline where applicable and filed the a priori protocol with the VA Evidence Synthesis Program Coordinating Center. Requirements for review and informed consent were waived because the study was designated as not human participants research.

### Literature Search

Literature searches were based on searches used for the evidence map of massage therapy completed in 2018.^8^ We searched 5 databases for relevant records published from July 2018 to April 2023 using the search terms “massage,” “acupressure,” “shiatsu,” “myofascial release therapy,” “systematic*,” “metaanaly*,” and similar terms. The databases were PubMed, the Allied and Complementary Medicine Database, the Cumulated Index to Nursing and Allied Health Literature, the Cochrane Database of Systematic Reviews, and Web of Science. See eAppendix 1 in Supplement 1 for full search strategies.

### Study Selection and Data Collection

Each title was screened independently by 2 authors for relevance (S.M., J.A., and P.G.S.). Abstracts were then reviewed in duplicate, with any discrepancies resolved by group discussion. To be included, abstracts or titles needed to be about efficacy or effectiveness of massage therapy for a painful adult health condition and be a systematic review with more than 1 study about massage therapy. A systematic review was defined as a review that had a documented systematic method for identifying and critically appraising evidence. In general, any therapist-delivered modality described as *massage therapy* by review authors was considered eligible (eg, tuina, acupressure, auricular acupressure, reflexology, and myofascial release). Sports massage therapy, osteopathy, dry cupping or dry needling, and internal massage therapy (eg, for pelvic floor pain) were ineligible, as were self-administered massage therapy techniques, like foam rolling. Reviews had to be about a painful condition for adults, and we excluded publications in low- and middle-income countries because of differences in resources for usual care or other active treatments for included conditions. Publications were required to compare massage therapy with sham or placebo massage, usual care, or other active therapies. Systematic reviews that covered other interventions were eligible if results for massage therapy were reported separately.

We next restricted eligibility to reviews that used formal methods to assess the certainty (sometimes called strength or quality) of the evidence for conclusions. In general, this meant using Grading of Recommendations, Assessment, Development, and Evaluations (GRADE).^9^ However, other formal methods were also included, such as the approach used by the US Agency for Healthcare Research and Quality (AHRQ) Evidence-based Practice Center (EPC) program. To be included, a review had to state or cite the method used and report the certainty (or strength or quality) of evidence for each conclusion. After we applied this restriction, most health conditions had only 1 systematic review meeting the eligibility criteria, and we used this review for the map. Among conditions for which we identified more than 1 review meeting the eligibility criteria, we first assessed whether reviews differed in some other feature used to classify reviews on our map (eg, different comparators or type of massage therapy), which we would label with the appropriate designation (such as *vs usual care* or *reflexology*). If there were multiple reviews about the same condition and they did not differ in some other feature, we selected the systematic review we judged as being most informative for readers. In general, this was the most recent review or the review with the greatest number of included studies.

### Data Extraction and Synthesis

Data on study condition, number of articles in a review, intervention characteristics, comparators, conclusions, and certainty, quality, or strength of evidence were extracted by 1 reviewer and then verified by a second reviewer (S.M., J.A., and P.G.S.). Our evidence mapping process produced a visual depiction of the evidence for massage therapy, as well as an accompanying narrative with an ancillary figure and table.

### Statistical Analysis

#### Evidence Map

The visual depiction or evidence map uses a bubble plot format to display information on 4 dimensions: bubble size, bubble label, x-axis, and y-axis. This allowed us to provide the following types of information about each included systematic review:

- **Number of articles in systematic review (bubble size):** The size of each bubble corresponds to the number of relevant primary research studies included in a systematic review.
- **Condition (bubble label):** Each bubble is labeled with the condition discussed by that systematic review.
- **Shapes and colors:** Intervention characteristics for each condition are presented in the form of colors (type of intervention) and shapes (comparators). For type of intervention, we included nonspecified massage therapy, tuina, myofascial release, reflexology, acupressure, and auricular acupressure. For comparators, we included mixed comparators with subgroups, mixed comparators with no subgroups, sham or placebo, and active therapy or usual care. A condition can appear more than once if multiple systematic reviews included different type of massage therapy or different comparators.
- **Strength of findings (rows):** Each condition is plotted on the map based on the ratings of certainty of evidence statement as reported in the systematic reviews: high, moderate, low, or very low.
- **Outcome associated with massage therapy (columns):** Each condition is plotted in potential benefit or no benefit as the outcome associated with massage therapy. Columns are not mutually exclusive. A review could have more than 1 conclusion, and conclusions could differ in the benefit associated with massage therapy. Both conclusions are included on the map.

#### Risk of Bias, Quality Assessment, and Certainty of Evidence

Risk of bias is not part of the method of an evidence map. We assessed the quality of included reviews using criteria developed by the U S Preventive Services Task Force (USPSTF). Certainty of evidence as determined by the original authors of the systematic review was abstracted for each conclusion in each systematic review and tabulated.

## Results

### Study Screening

**Figure 1.**
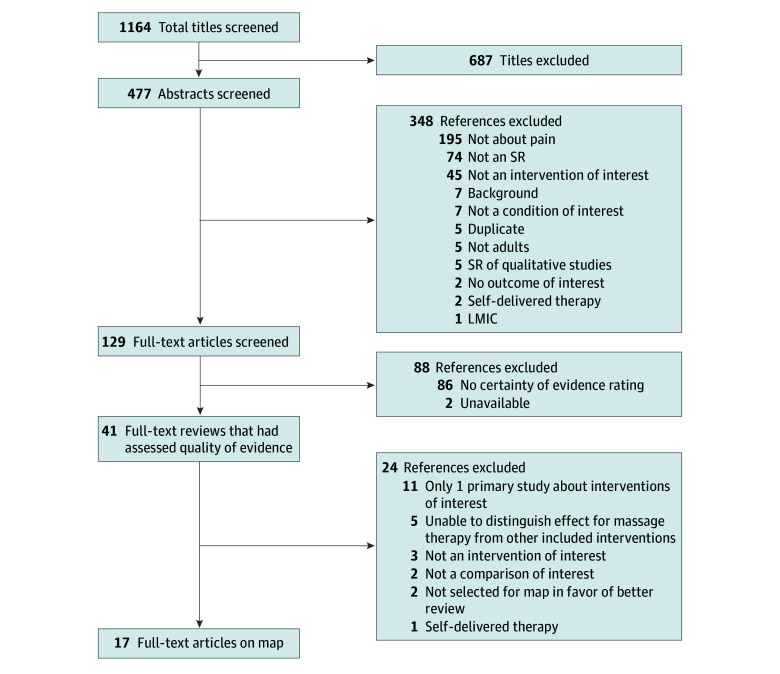
Literature Flowchart LMIC indicates low- and- middle-income country; SR, systematic review.

The search identified 1164 potentially relevant citations. Among 129 full-text articles screened, 41 publications were retained for further review. Of these, 24 reviews were excluded from the map for the following reasons: only 1 primary study about interventions of interest (11 studies), outcomes associated with massage therapy could not be distinguished from other included interventions (5 studies), not an intervention of interest (3 studies), not a comparison of interest (2 studies), overlap with a more recent or larger review that was already included on the map (2 studies), and self-delivered therapy (1 study). We included 17 publications in this map covering 13 health conditions.^4,10,11,12,13,14,15,16,17,18,19,20,21,22,23,24,25^ The literature flowchart (Figure 1) summarizes results of the study selection process, and eAppendix 2 in Supplement 1 presents citations for all excluded reviews at full-text screening.

### Study Characteristics

The total number of primary studies about massage therapy for pain in the included reviews ranged from 2 studies to 23 studies. There were 12 reviews that included fewer than 10 primary studies^4,11,12,13,14,15,16,17,20,21,22,23^ and 5 reviews that included 10 to 25 studies about massage therapy for pain.^10,18,19,24,25^ Of included reviews, 3 reviews were completed by the Cochrane Collaboration^4,19,23^ and 2 reviews were completed by the AHRQ EPC program.^11,18^

We categorized the included 17 reviews by health condition. These categories were cancer-related pain,^15,24^ back pain (including chronic back pain,^25^ chronic low back pain,^18,22^ and low back pain^17^), chronic neck pain,^18^ fibromyalgia,^21^ labor pain,^4,19^ mechanical neck pain,^13^ myofascial pain,^14^ palliative care needs,^10^ plantar fasciitis,^12^ post–breast cancer surgery pain,^16^ postcesarean pain,^23^ postpartum pain,^20^ and postoperative pain.^11^

Of 17 included reviews, 3 reviews included more than 1 type of massage therapy and 14 reviews included 1 type of massage therapy. Reviews by Chou et al^11^ and Smith et al^16^ included acupressure and nonspecified massage therapy as interventions. The review by Candy et al^7^ included reflexology and nonspecified massage therapy as interventions. Of the 14 reviews with 1 type of massage therapy, there were 5 reviews describing nonspecified massage therapy,^10,14,17,20^ 1 review about tuina,^22^ 5 reviews about myofascial release,^8,9,12,18,19^ and 3 reviews about acupressure.^13,15,21^

A variety of comparators were included in reviews. Of 9 reviews that included more than 1 comparator in analyses,^4,11,13,14,18,19,20,21,22^ 2 reviews did not conduct separate analyses by comparator (labeled *mixed with no subgroups*)^13,14^ and 3 reviews conducted separate analyses by comparator (labeled *mixed with subgroups*).^4,21,22^ The other 4 reviews included a mix of comparators with separate conclusions: sham or placebo and active therapy or usual care,^11^ mixed with no subgroups and active therapy or usual care,^18^ mixed with subgroups and active therapy or usual care,^20^ and mixed with no subgroups, sham, and active therapy or usual care.^19^ There were 8 reviews that included 1 comparator only in their analyses,^10,12,15,16,17,23,24,25^ with 7 reviews that described interventions compared with active therapy or usual care only,^10,12,15,17,23,24,25^ while 1 review limited inclusion to primary studies with a sham or placebo comparator.^16^

There was substantial variation in the reporting of other details from primary studies in included reviews. Any study that did not specify the mode of delivery was included; studies that explicitly stated that massage therapy was self-delivered were excluded. Of the 17 included reviews, 5 reviews provided details of personnel who administered the therapy, including massage therapist, nurse, aromatherapist, physiotherapist, and reflexologist.^4,10,19,20,21^ A total of 7 reviews presented length of sessions (eg, 5-minute or 90-minute sessions for massage therapy studies and 30-second or 5-minute sessions for acupressure studies).^10,16,18,20,21,22,23^ With the exception of the review by He et al,^15^ all reviews reported details about frequency, duration, or both when available. A total of 9 reviews included information about frequency of sessions (eg, 1 session or once every 3 weeks for massage therapy studies and 4 times per day or daily for acupressure studies),^10,12,16,17,18,20,21,22,23^ and 9 reviews reported duration of sessions (eg, single session or 3 months).^10,11,12,16,17,18,20,22,23^ There were 7 reviews that included details about follow-up (eg, 1 week or 12 months).^10,13,17,18,21,23,25^

### Quality Assessment

Using USPSTF criteria to rate the quality of included reviews, 10 reviews were rated good^4,10,11,14,15,16,18,19,21,23^ and 7 reviews were rated fair.^12,13,17,20,22,24,25^ See eAppendix 3 in Supplement 1 for each review’s rating.

### Evidence Map

**Figure 2.**
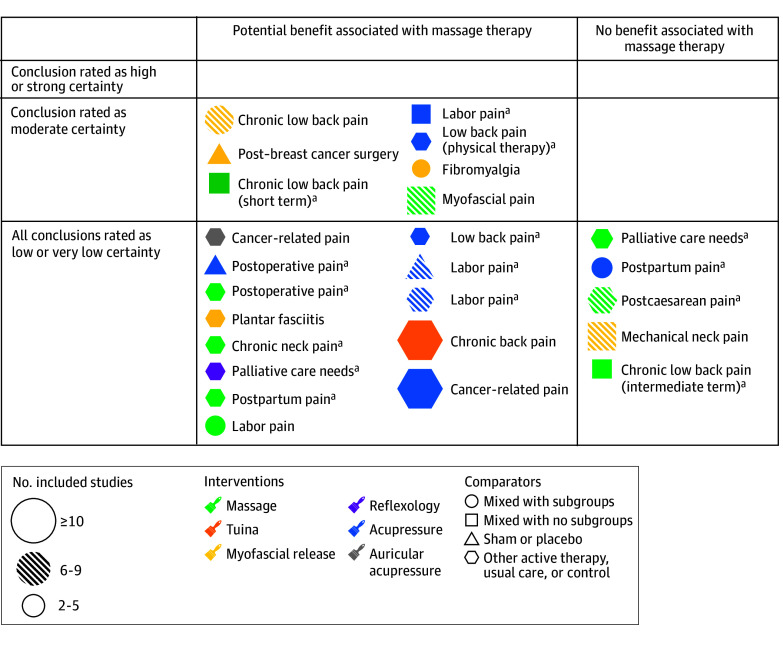
Evidence Map ^a^This review included distinct conclusions about separate conditions and comparators, and so it appears in this map more than once.

Figure 2 is a visual depiction of the following types of information about each included systematic review: condition, types of comparison treatments (shapes), types of massage therapy (color), number of articles included for each conclusion (bubble size), outcomes associated with massage therapy for pain (columns), and certainty of evidence rating (rows). There were 6 reviews mapped more than once, reflecting primary studies describing more than 1 health condition,^18^ more than 1 type of massage therapy,^10,20^ or outcomes associated with massage therapy compared with different comparators.^11,17,18,19^ There were 7 conditions from reviews^14,16,17,18,19,21,22^ that reported 1 conclusion rated as moderate-certainty evidence, all of which concluded that massage therapy was associated with beneficial outcomes for pain (Table 1). However, most other conditions had conclusions rated as low- or very low–certainty evidence (12 reviews about 10 conditions^4,10,11,12,13,15,17,18,19,20,23,24,25^). This rating means “Our confidence in the effect estimate is limited. The true effect may be substantially different from the estimate of effect,” or “We have very little confidence in the effect estimate.” See eAppendix 3 in Supplement 1 for conclusions in all reviews. This map included 4 conditions that did not appear in the 2018 map,^12,16,20,23^ and there were 8 conditions in the 2018 map that did not have new reviews meeting eligibility criteria (mainly a formal grading of the certainty of evidence); 7 health conditions^10,11,13,14,15,17,18,21,22,24,25^ were included in the 2018 map and the new map (see details in eAppendix 4 in Supplement 1).

**Table 1.** Certainty of Evidence Conclusions From Systematic Reviews Included in Evidence Map

Source	Condition	Conclusion as reported in original study
**Moderate certainty of evidence conclusions**		
Wu et al,^22^ 2021	Chronic low back pain	Compared with sham or active therapy, “myofascial release significantly improved pain” in patients with chronic low back pain.
Skelly et al,^18^ 2020	Chronic low back pain (short term)	Compared with sham or usual care, massage “showed small improvement…in pain short term” for chronic low back pain.
Ughreja et al,^21^ 2021	Fibromyalgia	Compared with sham or active therapy, myofascial release “significantly improve[d] pain…in patients with fibromyalgia syndrome.”
Smith et al,^19^ 2020	Labor pain	Compared with active therapy and routine care, there was evidence that “acupressure probably slightly reduces the intensity of pain during [labor].”
Li et al,^17^ 2021	Low back pain	Compared with active therapy (physical therapy), “moderate-quality evidence revealed an association between acupressure and greater pain relief” for low back pain.
Guzmán Pavón et al,^14^ 2022	Myofascial pain	Compared with no treatment, placebo, and active therapies, massage therapy has shown “a greater effect [on myofascial pain].”
Kannan et al,^16^ 2022	Post–breast cancer surgery	Compared with placebo, there were “positive treatment effects” for myofascial release on pain for post–breast cancer surgery pain.
**Low or very low certainty of evidence conclusions**		
He et al,^15^ 2020	Cancer-related pain	Compared with active therapy, auricular acupressure “was associated with significant reductions in pain intensity.”
Mai et al,^24^ 2022	Cancer-related pain	Compared with usual care, “acupressure combined with standard care may be associated with reduced pain intensity.”
Yang et al,^25^ 2023	Chronic back pain	Compared with active therapy, “Tuina might have a positive effect in reducing pain” in patients with chronic nonspecific low back pain.
Skelly et al,^18^ 2020	Chronic low back pain (intermediate term)	Compared with sham or usual care, massage showed “no difference…in intermediate-term [chronic low back] pain.”
Skelly et al,^18^ 2020	Chronic neck pain	Compared with attention or wait-list control, “massage conferred…a moderate improvement in pain short term” for chronic neck pain.
Smith et al,^19^ 2020	Labor pain	Compared with sham, “we are uncertain if acupressure reduces pain intensity in [labor].” Compared with usual care, “we are uncertain if acupressure reduces pain intensity in [labor].”
Smith et al,^4^ 2018	Labor pain	Compared with usual care, “massage provided a greater reduction in pain intensity…during the first stage of [labor].”
Li et al,^17^ 2021	Low back pain	Compared with active therapy or usual care, “acupressure could provide clinical benefits to [low back pain] conditions and had a significant short-term response rate in [low back pain] management.”
Guo et al,^13^ 2023	Mechanical neck pain	Compared with active therapy, “the differences were not significant to support [myofascial release] treatment on pain” for mechanical neck pain.
Candy et al,^10^ 2020	Palliative care needs	Compared with sham or active therapy, there was some evidence that “reflexology [reduced pain] for people with palliative care needs.” Compared with active therapy, there was “no evidence of short-term benefits of massage on…pain for people with palliative care needs.”
Guimarães et al,^12^ 2022	Plantar fasciitis	Compared with “control in the short term,” myofascial release “resulted in effective treatment for pain” for plantar fasciitis.
Zimpel et al,^23^ 2020	Postcesarean pain	Compared with active therapy, “we are uncertain if hand and foot massage therapy plus analgesia…has any effect on [postcaesarean] pain.”
Chou et al,^11^ 2020	Postoperative pain	Compared with sham, acupressure is effective for postoperative pain. Compared with active therapy, “there was low strength of evidence supporting effectiveness of massage for postoperative pain.”
Smith et al,^20^ 2022	Postpartum pain	Compared with active or routine care, there was “a reduction in pain [from massage therapy] following caesarean birth.” Compared with sham or routine care, “acupressure…found no improvement in postpartum pain management.”

### Adverse Events

Evidence about adverse events was collected by approximately half of included reviews, and no serious adverse events were reported. While 11 of 17 reviews^10,11,13,15,17,18,19,22,23,24,25^ described adverse events, 2 reviews^18,23^ included certainty of evidence conclusions for adverse events for 3 health conditions (Table 2).

**Table 2.** Certainty of Evidence Conclusions of Adverse Events From Systematic Reviews in Evidence Map

Source	Condition	Description of adverse events	Conclusion as reported in original study	Certainty of evidence
Zimpel et al,^23^ 2020	Postcesarean pain	“Anxiety assessed by different scores with the use of massage therapy at 90 minutes after the intervention and at 60 minutes after the intervention.”	“It is uncertain if massage plus analgesia has any effect on anxiety compared with analgesia alone at 90 minutes.”	Very low
Skelly et al,^18^ 2020	Low back pain	<1%-26% Of participants reported additional pain after receiving massage therapy	“Four trials reported no serious adverse events, and one trial reported no adverse events.”	Low
Skelly et al,^18^ 2020	Neck pain	Mild adverse effects, such as discomfort or pain during or after Swedish massage therapy, increased pain after Tuina, or “dizziness, sleepiness, mood swings, nausea, difficulty staying asleep, difficulty moving the head and neck.”	“No evidence of increased risk of serious harms.”	Low

## Discussion

There is a large literature of original randomized clinical trials and systematic reviews of randomized clinical trials of massage therapy as a treatment for pain. Our systematic review found that despite this literature, there were only a few conditions for which authors of systematic reviews concluded that there was at least moderate-certainty evidence regarding health outcomes associated with massage therapy and pain. Most reviews reported low- or very low–certainty evidence. Although adverse events associated with massage therapy for pain were rare, the evidence was limited. For reviews that had conclusions about adverse events, authors were uncertain if there was a difference between groups or did not find a difference between groups and rated the evidence low to very low certainty of evidence.

Massage therapy is a broad term that is inclusive of many styles and techniques. We applied exclusion criteria determined a priori to help identify publications for inclusion in the evidence map. Despite that procedure, there was still a lack of clarity in determining what massage therapy is. For instance, acupressure was sometimes considered acupuncture and other times considered massage therapy, depending on author definition. In this case, we reviewed and included only publications that were explicitly labeled acupressure and did not review publications about acupuncture only. This highlights a fundamental issue with examining the evidence base of massage therapy for pain when there is ambiguity in defining what is considered massage therapy.

Unlike a pharmaceutical placebo, sham massage therapy may not be truly inactive. It is conceivable that even the light touch or touch with no clear criterion^26^ used in sham massage therapy may be associated with some positive outcomes, meaning that patients who receive the massage therapy intervention and those who receive a sham massage therapy could both demonstrate some degree of symptom improvement. Limitations of sham comparators raise the question of whether sham or placebo treatment is an appropriate comparison group in massage therapy trials. It may be more informative to compare massage therapy with other treatments that are accessible and whose benefits are known so that any added beneficial outcomes associated with massage therapy could be better isolated and understood.

Compared with the 2018 map, our map included 4 new conditions not on the 2018 map, while 8 conditions from the 2018 map had no new reviews meeting eligibility criteria and 7 health conditions appeared in both maps. Despite identifying new conditions and conclusions with higher certainty of evidence in several reviews in our updated search, most included reviews reported low or very low certainty of evidence, suggesting that the most critical research need is for better evidence to increase certainty of evidence for massage therapy for pain. This is a challenge given that massage, like other complementary and integrative health interventions, does not have the historical research infrastructure that most health professions have.^27^ Nevertheless, it is only when systematic reviews and meta-analyses are conducted with high-quality primary studies that the association or lack of association of massage therapy with pain will reach higher certainties of evidence. Studies comparing massage therapy with placebo or sham are probably not the priority; rather, the priority should be studies comparing massage therapy with other recommended, accepted, and active therapies for pain. Studies comparing massage therapy with other recommended therapies should also have a sufficiently long follow-up to allow any nonspecific outcomes (eg, those associated with receiving some new treatment) to dissipate. For example, this period has been proposed to be at least 6 months for studies of chronic pain.

### Limitations

There are 2 main limitations to this systematic review’s evidence map. The first, common to all systematic reviews, is that we may not have identified all potentially eligible evidence. If a systematic review was published in a journal not indexed in any of 5 databases we searched and we did not identify it as part of our search of references of included publications, then we would have missed it. Nevertheless, our search strategy identified more than 200 publications about massage therapy for pain published since July 2018, so we did not lack potential reviews to evaluate. The second limitation of evidence maps is that we did not independently evaluate the source evidence; in other words, we took conclusions of authors of the systematic review at face value. That is the nature of an evidence map. Particular to this application of the mapping process, we mapped the review we deemed most informative for the 2 health conditions that had more than 1 eligible review (back pain and labor pain). This necessarily requires judgment, and others could disagree with that judgment. We included the citation for reviews excluded from the map for this overlap reason in supplemental material, and interested readers can review it for additional information. As in all evidence-based products and particularly in 1 such as this covering a large and complex evidence base, it is possible that there are errors of data extraction and compilation. We used dual review to minimize the chance of such errors, but if we are notified of errors, we will correct them.

## Conclusions

Although this systematic review found that the number of conclusions about the effectiveness of massage therapy that were judged to have at least moderate certainty of evidence was greater now than in 2018, it was still small relative to the need. More high-quality randomized clinical trials are needed to provide a stronger evidence base to assess the effect of massage therapy on pain. For painful conditions that do not have at least moderate-certainty evidence supporting use of massage therapy, new studies that address limitations of existing research are needed. The field of massage therapy would be best advanced by educating the wider research community with clearer definitions of massage therapy and whether it is appropriate to include multiple modalities in the same systematic review.

## Notes

Supplement 1. **eAppendix 1.** Search Strategies **eAppendix 2.** Excluded Studies **eAppendix 3.** Evidence Table **eAppendix 4.** Conditions in Previously Published Map in 2018 and Current Map
eReferences.

Supplement 2.
Data Sharing Statement
