# Supplementary material for: Use of Massage Therapy for Pain, 2018-2023: A Systematic Review
Source: JAMA Netw Open. 2024 Jul 15;7(7):e2422259. doi: 10.1001/jamanetworkopen.2024.22259 (PMC11250267; doi:10.1001/jamanetworkopen.2024.22259)
Supplement: Supplement 1. — eAppendix 1. Search Strategies eAppendix 2. Excluded Studies eAppendix 3. Evidence Table eAppendix 4. Conditions in Previously Published Map in 2018 and Current Map eReferences. [file jamanetwopen-e2422259-s001.pdf]

Supplemental Online Content

Mak S, Allen J, Begashaw M, et al. Use of massage therapy for pain, 2018-2023: a systematic review. *JAMA Netw Open*. 2024;7(7):e2422259.
doi:10.1001/jamanetworkopen.2024.22259

eAppendix 1. Search Strategies
eAppendix 2. Excluded Studies
eAppendix 3. Evidence Table
eAppendix 4. Conditions in Previously Published Map in 2018 and Current Map
eReferences.

This supplemental material has been provided by the authors to give readers additional information about their work.

© 2024 Mak S et al. *JAMA Network Open.*

Appendix 1. Search Strategies

PubMed
7/10/2018 – 12/31/2022; English Language
massag*[tiab] OR shiatsu[tiab] OR "myofascial"[tiab] OR acupressure*[tiab] OR Massage[Mesh] OR Acupressure[Mesh] OR "Myofascial Release Therapy"[Mesh]
AND
(systematic*[tiab] AND review[tiab]) OR "systematic overview*"[tiab] OR "Cochrane review*"[tiab] OR "systemic review*"[tiab] OR meta-analy*[tiab] OR metaanaly*[tiab] OR "meta analysis"[tiab] OR meta-review[tiab] OR meta-synthesis[tiab] OR "meta synthesis"[tiab] OR metasynthesis[tiab] OR "quantitative review"[tiab] OR "quantitative synthesis"[tiab] OR meta-analy*[tiab] OR (meta-analytic*[tiab] AND review[tiab]) OR meta-analysis[pt] OR "systematic review"[pt] OR “systematic review*”[tiab]
Results: **709**

CINAHL
07/2018 – 12/2023; English; Academic Journals
((TI massag* OR AB massag*) OR (TI shiatsu OR AB shiatsu) OR (TI myofascial OR AB myofascial) OR (TI acupressure* OR AB acupressure*) OR (MH "Massage+") OR (MH Acupressure+) OR (MH "Myofascial Release"))
AND
((TI systematic* OR AB systematic*) AND (TI review OR AB review)) OR (TI "systematic overview*" OR AB "systematic overview*") OR (TI "Cochrane review*" OR AB "Cochrane review*") OR (TI "systemic review*" OR AB "systemic review*") OR (TI meta-analy* OR AB meta-analy*) OR (TI metaanaly* OR AB metaanaly*) OR (TI "meta analysis" OR AB "meta analysis") OR (TI meta-review OR AB meta-review) OR (TI meta-synthesis OR AB meta-synthesis) OR (TI "meta synthesis" OR AB "meta synthesis") OR (TI metasynthesis OR AB metasynthesis) OR (TI "quantitative review" OR AB "quantitative review") OR (TI "quantitative synthesis" OR AB "quantitative synthesis") OR ((TI meta-analytic* OR AB meta-analytic*) AND (TI review OR AB review)) OR (TI "systematic review*" OR AB "systematic review*") OR (MH "Systematic Review")
Results: **135**

Cochrane Database of Systematic Reviews
Filters: 07/2018 – 12/2023; Excluding Trials
((massag* OR shiatsu OR myofascial OR acupressure*):ti,ab OR [mh Massage] OR [mh Acupressure] OR [mh "Myofascial Release Therapy"])
Results: **19**

© 2024 Mak S et al. *JAMA Network Open.*

Web of Science
Filters: 2018-2023; English
(TS=massag* OR TS=shiatsu OR TS=myofascial OR TS=acupressure*)
AND
((TI=systematic* OR AB=systematic*) AND (TI=review OR AB=review)) OR (TI="systematic overview*" OR AB="systematic overview*") OR (TI="Cochrane review*" OR AB="Cochrane review*") OR (TI="systemic review*" OR AB="systemic review*") OR (TI=meta-analy* OR AB=meta-analy*) OR (TI=metaanaly* OR AB=metaanaly*) OR (TI="meta analysis" OR AB="meta analysis") OR (TI=meta-review OR AB=meta-review) OR (TI=meta-synthesis OR AB=meta-synthesis) OR (TI="meta synthesis" OR AB="meta synthesis") OR (TI=metasynthesis OR AB=metasynthesis) OR (TI="quantitative review" OR AB="quantitative review") OR
(TI="quantitative synthesis" OR AB="quantitative synthesis") OR ((TI=meta-analytic* OR AB=meta-analytic*) AND (TI=review OR AB=review)) OR (TI="systematic review*" OR AB="systematic review*")
Results: **297**

AMED
Filters: 07/2018 – 12/2022; English; Academic Journals
((TI massag* OR AB massag*) OR (TI shiatsu OR AB shiatsu) OR (TI myofascial OR AB myofascial) OR (TI acupressure* OR AB acupressure*) OR (DE "MASSAGE") OR (DE "ACUPRESSURE"))
AND
((TI systematic* OR AB systematic*) AND (TI review OR AB review)) OR (TI "systematic overview*" OR AB "systematic overview*") OR (TI "Cochrane review*" OR AB "Cochrane review*") OR (TI "systemic review*" OR AB "systemic review*") OR (TI meta-analy* OR AB meta-analy*) OR (TI metaanaly* OR AB metaanaly*) OR (TI "meta analysis" OR AB "meta analysis") OR (TI meta-review OR AB meta-review) OR (TI meta-synthesis OR AB meta-synthesis) OR (TI "meta synthesis" OR AB "meta synthesis") OR (TI metasynthesis OR AB metasynthesis) OR (TI "quantitative review" OR AB "quantitative review") OR (TI "quantitative synthesis" OR AB "quantitative synthesis") OR ((TI meta-analytic* OR AB meta-analytic*) AND (TI review OR AB review)) OR (TI "systematic review*" OR AB "systematic review*")
Results: **4**

© 2024 Mak S et al. *JAMA Network Open.*

Appendix 2. Excluded Studies

<u>Only One Primary Study About Interventions of Interest, *N = 11*</u>

1. Anderson, L., et al., Effectiveness of breast massage for the treatment of women with breastfeeding problems: a systematic review. JBI Database System Rev Implement Rep, 2019. 17(8): p. 1668-1694.
2. Asquini, G., et al., Effectiveness of manual therapy applied to craniomandibular structures in temporomandibular disorders: A systematic review. Journal of Oral Rehabilitation, 2022. 49(4): p. 442-455.
3. Crepinsek, M.A., et al., Interventions for preventing mastitis after childbirth. Cochrane Database Syst Rev, 2020. 9(9): p. Cd007239.
4. Fan, Z., et al., The effectiveness and safety of Tuina for tension-type headache: A systematic review and meta-analysis. Complementary Therapies in Clinical Practice, 2021. 43: p. N.PAG-N.PAG.
5. Franco, J.V.A., et al., Non-pharmacological interventions for treating chronic prostatitis/chronic pelvic pain syndrome: a Cochrane systematic review. BJU Int, 2019. 124(2): p. 197-208.
6. Hadoush, H., et al., Effectiveness of non-pharmacological rehabilitation interventions in pain management in patients with multiple sclerosis: Systematic review and meta-analysis. NeuroRehabilitation, 2022. 50(4): p. 347-365.
7. Huisstede, B.M., et al., Carpal Tunnel Syndrome: Effectiveness of Physical Therapy and Electrophysical Modalities. An Updated Systematic Review of Randomized Controlled Trials. Arch Phys Med Rehabil, 2018. 99(8): p. 1623-1634.e23.
8. Juncker, R.B., F.M. Mirza, and J.J. Gagnier, Reduction in opioid use with perioperative non-pharmacologic analgesia in total knee arthroplasty and ACL reconstruction: a systematic review. Sicot j, 2021. 7: p. 63.
9. López-Liria, R., et al., Efficacy of Physiotherapy Treatment in Primary Dysmenorrhea: A Systematic Review and Meta-Analysis. Int J Environ Res Public Health, 2021. 18(15).
10. Schulze, N.B., et al., Efficacy of Manual Therapy on Pain, Impact of Disease, and Quality of Life in the Treatment of Fibromyalgia: A Systematic Review. Pain Physician, 2020. 23(5): p. 461-476.
11. Zakarija-Grkovic, I. and F. Stewart, Treatments for breast engorgement during lactation. Cochrane Database Syst Rev, 2020. 9(9): p. Cd006946.

<u>Unable to Distinguish Effect for Massage Therapy from Other Included Interventions, *N* = 5</u>

1. Denneny, D., et al., Trigger Point Manual Therapy for the Treatment of Chronic Noncancer Pain in Adults: A Systematic Review and Meta-analysis. Archives of Physical Medicine and Rehabilitation, 2019. 100(3): p. 562-577.
2. Jung, A., et al., Effectiveness of physiotherapy interventions on headache intensity, frequency, duration and quality of life of patients with tension-type headache. A systematic review and network meta-analysis. Cephalalgia, 2022. 42(9): p. 944-965.
3. Kamonseki, D.H., et al., Effectiveness of manual therapy in patients with tension-type headache. A systematic review and meta-analysis. Disability and Rehabilitation, 2022. 44(10): p. 1780-1789.

© 2024 Mak S et al. *JAMA Network Open.*

4. Qin, D., et al., Acupuncture and related techniques for postoperative pain after hemorrhoidectomy: A systematic review and network meta-analysis. Eur J Integr Med, 2020. 37.
5. Wu, Q., J. Zhao, and W. Guo, Efficacy of massage therapy in improving outcomes in knee osteoarthritis: A systematic review and meta-analysis. Complement Ther Clin Pract, 2022. 46: p. 101522.

Not an Intervention of Interest, *N = 3*

1. Effects of Myofascial Manipulative Therapies in Chronic Pelvic Pain Syndromes: A Systematic Review and Meta-Analysis. Journal of Bodywork & Movement Therapies, 2023. 33: p. e92-e92.
2. Dal Farra, F., et al., Effectiveness of Myofascial Manual Therapies in Chronic Pelvic Pain Syndrome: A Systematic Review and Meta-Analysis. Int Urogynecol J, 2022. 33(11): p. 2963-2976.
3. Fuentes-Márquez, P., I. Cabrera-Martos, and M.C. Valenza, Physiotherapy interventions for patients with chronic pelvic pain: A systematic review of the literature. Physiother Theory Pract, 2019. 35(12): p. 1131-1138.

Not a Comparison of Interest, *N = 2*

1. Lavazza, C., et al., Sham treatment effects in manual therapy trials on back pain patients: a systematic review and pairwise meta-analysis. BMJ Open, 2021. 11(5): p. e045106.
2. Mo, Z., et al., Comparisons of the Effectiveness and Safety of Tuina, Acupuncture, Traction, and Chinese Herbs for Lumbar Disc Herniation: A Systematic Review and Network Meta-Analysis. Evidence-based Complementary & Alternative Medicine (eCAM), 2019: p. 1-10.

Review Meeting Eligibility Criteria but Not Included in Evidence Map in Favor of a Better Review, *N = 2*

1. Chen, Z., et al., The effects of myofascial release technique for patients with low back pain: A systematic review and meta-analysis. Complement Ther Med, 2021. 59: p. 102737.
2. Li, Y., et al., Effects of perineal massage during childbirth on maternal and neonatal outcomes in primiparous women: A systematic review and meta-analysis. Int J Nurs Stud, 2022. 138: p. 104390.

Self-Delivered Therapy, *N = 1*

1. Cao, X., et al., PFMT relevant strategies to prevent perineal trauma: a systematic review and network meta-analysis. Arch Gynecol Obstet, 2022.

© 2024 Mak S et al. *JAMA Network Open.*

Appendix 3. Evidence Table

Author, Year<br>Country	Condition	Type of Massage Therapy<br>Comparator<br># of Included Primary Studies About Massage Therapy	Certainty of Evidence Rating	Conclusion as Reported in Original Paper	Quality of Review
He, 2020[1]<br>China, Australia	Cancer-related pain	Auricular acupressure<br>Active therapy/usual care/control<br>2	Low or Very Low	Compared with active therapy, auricular acupressure "was associated with significant reductions in pain intensity."	Good
Mai, 2022[2]<br>Iran, China	Cancer-related pain	Acupressure<br>Active therapy/usual care/control<br>23	Low or Very Low	Compared with usual care, "acupressure combined with standard care may be associated with reduced pain intensity."	Fair
Yang, 2023[3]<br>China, Korea, USA	Chronic back pain	Tuina<br>Active therapy/usual care/control<br>12	Low or Very Low	Compared with active therapy, "Tuina might have a positive effect in reducing pain" in patients with chronic non-specific low back pain.	Fair
Wu, 2021[4]<br>China	Chronic low back pain	Myofascial release<br>Mixed with subgroups<br>8	Moderate	Compared with sham or active therapy, "myofascial release significantly improved pain" in patients with chronic low back pain.	Fair
Skelly, 2020[5]<br>USA	Chronic low back pain	Massage therapy<br>Mixed with no subgroups<br>5	Moderate	Compared with sham or usual care, massage "showed small improvement…in pain short term" for chronic low back pain.	Good

© 2024 Mak S et al. *JAMA Network Open.*

Author, Year Country	Condition	Type of Massage Therapy<br>Comparator<br># of Included Primary Studies About Massage Therapy	Certainty of Evidence Rating	Conclusion as Reported in Original Paper	Quality of Review
		Massage therapy<br>Mixed with no subgroups<br>3	Low or Very Low	Compared with sham or usual care, massage showed "no difference…in intermediate-term [chronic low back] pain."	
	Chronic neck pain	Massage therapy<br>Active therapy/usual care/control<br>3	Low or Very Low	Compared with attention or waitlist control, "massage conferred…a moderate improvement in pain short term" for chronic neck pain.	
Ughreja, 2021[6]<br>India	Fibromyalgia	Myofascial release<br>Mixed with subgroups<br>2	Moderate	Compared with sham or active therapy, myofascial release "significantly improve[d] pain…in patients with fibromyalgia syndrome."	Good
Smith, 2018[7]<br>Australia	Labor pain	Massage therapy<br>Mixed with subgroups<br>4	Low or Very Low	Compared with usual care, "massage provided a greater reduction in pain intensity…during the first stage of [labor]."	Good
Smith, 2020[8]<br>Australia, Singapore, Iran	Labor pain	Acupressure<br>Sham<br>6	Low or Very Low	Compared with sham, "we are uncertain if acupressure reduces pain intensity in [labor]."	Good
		Acupressure<br>Active therapy/usual care/control	Low or Very Low	Compared with usual care, "we are uncertain if acupressure reduces pain intensity in [labor]."	

© 2024 Mak S et al. *JAMA Network Open.*

Author, Year Country	Condition	Type of Massage Therapy Comparator # of Included Primary Studies About Massage Therapy	Certainty of Evidence Rating	Conclusion as Reported in Original Paper	Quality of Review
		8			
		Acupressure<br>Mixed no subgroups<br>2	Moderate	Compared with active therapy and routine care, there was evidence that "acupressure probably slightly reduces the intensity of pain during [labor]."	
Li, 2021[9]<br>China	Low back pain	Acupressure<br>Active therapy/usual care/control<br>3	Moderate	Compared with active therapy (physical therapy), "moderate-quality evidence revealed an association between acupressure and greater pain relief" for low back pain.	Fair
		Acupressure<br>Active therapy/usual care/control<br>2	Low or Very Low	Compared with active therapy or usual care, "acupressure could provide clinical benefits to [low back pain] conditions and had a significant short-term response rate in [low back pain] management."	
Guo, 2023[10]<br>China	Mechanical neck pain	Myofascial release<br>Mixed with no subgroups<br>9	Low or Very Low	Compared with active therapy and sham, "the differences were not significant to support [myofascial release] treatment on pain" for mechanical neck pain.	Fair

© 2024 Mak S et al. *JAMA Network Open.*

Author, Year Country	Condition	Type of Massage Therapy<br>Comparator<br># of Included Primary Studies About Massage Therapy	Certainty of Evidence Rating	Conclusion as Reported in Original Paper	Quality of Review
Guzmán Pavón, 2022[11]<br>Spain, Chile, Paraguay	Myofascial pain	Massage therapy<br>Mixed no subgroups<br>8	Moderate	Compared with no treatment, placebo, and active therapies, massage therapy has shown "a greater effect [on myofascial pain]."	Good
Candy, 2020[12]<br>England	Palliative care needs	Reflexology<br>Active therapy/usual care/control<br>3	Low or Very Low	Compared with sham or active therapy, there was some evidence that "reflexology [reduced pain] for people with palliative care needs."	Good
		Massage therapy<br>Active therapy/usual care/control<br>5	Low or Very Low	Compared with active therapy, there was "no evidence of short-term benefits of massage on … pain for people with palliative care needs."	
Guimarães, 2022[13]<br>Brazil	Plantar fasciitis	Myofascial release<br>Active therapy/usual care/control<br>4	Low or Very Low	Compared with "control in the short term", myofascial release "resulted in effective treatment for pain" for plantar fasciitis.	Fair
Kannan, 2022[14]<br>China	Post-breast cancer surgery	Myofascial release<br>Sham/placebo<br>2	Moderate	Compared with placebo, there were "positive treatment effects" for myofascial release on pain for post-breast cancer surgery pain.	Good

© 2024 Mak S et al. *JAMA Network Open.*

Author, Year Country	Condition	Type of Massage Therapy<br>Comparator<br># of Included Primary Studies About Massage Therapy	Certainty of Evidence Rating	Conclusion as Reported in Original Paper	Quality of Review
Zimpel, 2020[15]<br>Brazil	Post-caesarean pain	Massage therapy<br>Active therapy/usual care/control<br>9	Low or Very Low	Compared with active therapy, "we are uncertain if hand and foot massage therapy plus analgesia…has any effect on [post-caesarean] pain."	Good
Chou, 2020[16]<br>USA	Post-operative pain	Acupressure<br>Sham/placebo<br>2	Low or Very Low	Compared with sham, acupressure is effective for post-operative pain.	Good
		Massage therapy<br>Active therapy/usual care/control<br>5	Low or Very Low	Compared with active therapy, "there was low strength of evidence supporting effectiveness of massage for postoperative pain."	
Smith, 2022[17]<br>Australia	Post-partum pain	Massage therapy<br>Active therapy/usual care/control<br>4	Low or Very Low	Compared with active or routine care, there was "a reduction in pain [from massage therapy] following caesarean birth."	Fair
		Acupressure<br>Mixed with subgroups<br>2	Low or Very Low	Compared with sham or routine care, "acupressure…found no improvement in postpartum pain management."	

© 2024 Mak S et al. *JAMA Network Open.*

Appendix 4. Conditions in Previously Published Map in 2018 and Current Map

We mapped conclusion(s) for the effect of massage therapy on the conditions that were also included in the previous evidence map: back pain (including chronic low back pain and chronic back pain), cancer-related pain, fibromyalgia, myofascial pain, neck pain (including chronic neck pain and mechanical neck pain), palliation-related pain, and post-operative pain (Table A1).

Table A1. Conditions in Both 2018 and 2023 Evidence Maps

Condition	Primary Studies Published Since 2018	# of Reviews for the Condition
Back pain (including chronic low back pain and chronic back pain)	16	4
Cancer-related pain	13	2
Fibromyalgia	0	1
Myofascial pain	3	1
Neck pain (including chronic neck pain and mechanical neck pain)	0	2
Palliation-related pain	1	1
Post-operative pain	2	1

We surveyed the publication year of the primary studies included in the 11 reviews about these 7 conditions to assess the number of new trials published in or after 2018. With the exception of fibromyalgia,[6] the other 6 conditions were represented by reviews which had included primary studies that were published in 2018 or after. For back pain, 16 studies from 3 reviews were published since 2018: myofascial release for chronic back pain ($N = 4$),[4] Tuina for chronic back pain ($N = 7$),[3] and acupressure for low back pain ($N = 5$).[9] A fourth review about the short and intermediate-term effect of massage therapy for chronic low back pain did not include trials published since 2018.[5] For cancer-related pain, 13 studies about acupressure from 1 review were published since 2018.[2] No primary studies contributing to the potential benefit of auricular acupressure for cancer-related pain were published prior to 2018.[1] For myofascial pain, 3 studies about massage therapy from 1 review were published since 2018.[11] For neck pain, 7 studies about myofascial release from 1 review were published since 2018.[10] A second review about massage therapy for neck pain did not include trials published since 2018.[5] For palliation-related pain, 1 review had included 1 primary study about reflexology which was published in 2018.[12] For post-operative pain, 2 reviews about massage therapy were published since 2018.[16]

This map includes 4 conditions that were not part of the previous map (Table A2).

© 2024 Mak S et al. *JAMA Network Open.*

Table A2. Newly Identified Conditions in 2023 Evidence Map

Condition	Primary Studies Published Since 2018
Plantar fasciitis	2
Post-breast cancer surgery	1
Post-caesarean pain	2
Post-partum pain	2

Eleven conditions were included the previous map but are not in the current report: arthritis, cervical radiculopathy, dysmenorrhea, elbow pain, headache, labor pain, mixed musculoskeletal pain, muscle soreness, pain in critical care setting, scar pain, shoulder pain, and temporomandibular joint pain. We did not identify citations about arthritis, cervical radiculopathy, or muscle soreness in the update search to be reviewed at full text. Table A3 provides details for the other 8 conditions.

Because we applied an additional criterion that reviews had to report a method used for grading certainty of evidence to be included in this map, a few conditions that had appeared in the previous map were not included in this map. Some publications were excluded due to only including 1 primary study with an intervention of interest; 1 publication did not include pain as an outcome of interest; and other reviews did not separate the analysis of the effect of different treatments for a condition, which means we were unable to distinguish the effect for massage therapy from other included interventions.

Table A3. Selected Conditions in Previous Evidence Map Not in 2023 Evidence Map

Condition	New Systematic Review Identified in Update Search?	Exclusion Reason
Arthritis	No	N/A
Cervical radiculopathy	No	N/A
Critical care	1	• No certainty of evidence rating (*N* = 1)
Dysmenorrhea	5	• No certainty of evidence rating (*N* = 4)<br>• Included only 1 primary study of interest (*N* = 1)
Elbow	1	• No certainty of evidence rating (*N* = 1)
Headache	5	• No certainty of evidence rating (*N* = 2)<br>• Included only 1 primary study of interest (*N* = 1)<br>• 2 not separate
Labor	3	• No certainty of evidence rating (*N* = 1)
Mixed musculoskeletal		
Knee osteoarthritis	7	• No certainty of evidence rating (*N* = 6)<br>• Unable to distinguish effect for massage therapy from other included interventions (*N* = 1)

© 2024 Mak S et al. *JAMA Network Open.*

Condition	New Systematic Review Identified in Update Search?	Exclusion Reason
Carpal tunnel syndrome	1	• Included only 1 primary study of interest (*N* = 1)
Acute/chronic	3	• No certainty of evidence rating (*N* = 3)
Muscle soreness	No	N/A
Scar	3	• No certainty of evidence rating (*N* = 1)<br>• No outcome of interest (*N* = 1)
Shoulder	1	• No certainty of evidence rating (*N* = 1)
Temporomandibular joint	2	• No certainty of evidence rating (*N* = 1)<br>• Included only 1 primary study of interest (*N* = 1)

© 2024 Mak S et al. *JAMA Network Open.*

References

1. He Y, Guo X, May BH, et al. Clinical Evidence for Association of Acupuncture and Acupressure With Improved Cancer Pain: A Systematic Review and Meta-Analysis. *JAMA Oncol*. Feb 1 2020;6(2):271-278. doi:10.1001/jamaoncol.2019.5233
2. Mai QL, Li XJ, Yang D, Zhang XY, Peng K, Hao YF. Effects of acupressure on cancer-related pain management: A systematic review and meta-analysis of randomized controlled trials. Article. *Eur J Integr Med*. Apr 2022;51:9. 102120. doi:10.1016/j.eujim.2022.102120
3. Yang J, Zhou X, Ma QY, et al. Efficacy and safety of Tuina for chronic nonspecific low back pain: A PRISMA-compliant systematic review and meta-analysis. Review. *Medicine*. Mar 2023;102(9):11. doi:10.1097/md.0000000000033018
4. Wu Z, Wang Y, Ye X, et al. Myofascial Release for Chronic Low Back Pain: A Systematic Review and Meta-Analysis. *Front Med (Lausanne)*. 2021;8:697986. doi:10.3389/fmed.2021.697986
5. Skelly AC, Chou R, Dettori JR, et al. Noninvasive Nonpharmacological Treatment for Chronic Pain: A Systematic Review Update: AHRQ Comparative Effectiveness Reviews. Agency for Healthcare Research and Quality (US); 2020.
6. Ughreja RA, Venkatesan P, Balebail Gopalakrishna D, Singh YP. Effectiveness of myofascial release on pain, sleep, and quality of life in patients with fibromyalgia syndrome: A systematic review. *Complement Ther Clin Pract*. Nov 2021;45:101477. doi:10.1016/j.ctcp.2021.101477
7. Smith CA, Levett KM, Collins CT, Dahlen HG, Ee CC, Suganuma M. Massage, reflexology and other manual methods for pain management in labour. *Cochrane Database Syst Rev*. Mar 28 2018;3(3):Cd009290. doi:10.1002/14651858.CD009290.pub3
8. Smith CA, Collins CT, Levett KM, et al. Acupuncture or acupressure for pain management during labour. *Cochrane Database Syst Rev*. Feb 7 2020;2(2):Cd009232. doi:10.1002/14651858.CD009232.pub2
9. Li T, Li X, Huang F, Tian Q, Fan ZY, Wu S. Clinical Efficacy and Safety of Acupressure on Low Back Pain: A Systematic Review and Meta-Analysis. *Evid Based Complement Alternat Med*. 2021;2021:8862399. doi:10.1155/2021/8862399
10. Guo Y, Lv X, Zhou Y, et al. Myofascial release for the treatment of pain and dysfunction in patients with chronic mechanical neck pain: Systematic review and meta-analysis of randomised controlled trials. *Clin Rehabil*. Oct 27 2022:2692155221136108. doi:10.1177/02692155221136108
11. Guzmán Pavón MJ, Cavero Redondo I, Martínez Vizcaíno V, Ferri Morales A, Lorenzo García P, Álvarez Bueno C. Comparative Effectiveness of Manual Therapy Interventions on Pain and Pressure Pain Threshold in Patients With Myofascial Trigger Points: A Network Meta-analysis. *Clin J Pain*. Dec 1 2022;38(12):749-760. doi:10.1097/ajp.0000000000001079
12. Candy B, Armstrong M, Flemming K, et al. The effectiveness of aromatherapy, massage and reflexology in people with palliative care needs: A systematic review. *Palliat Med*. Feb 2020;34(2):179-194. doi:10.1177/0269216319884198
13. Guimarães JS, Arcanjo FL, Leporace G, et al. Effects of therapeutic interventions on pain due to plantar fasciitis: A systematic review and meta-analysis. *Clin Rehabil*. Dec 26 2022:2692155221143865. doi:10.1177/02692155221143865
14. Kannan P, Lam HY, Ma TK, Lo CN, Mui TY, Tang WY. Efficacy of physical therapy interventions on quality of life and upper quadrant pain severity in women with post-mastectomy

© 2024 Mak S et al. *JAMA Network Open.*

pain syndrome: a systematic review and meta-analysis. *Qual Life Res*. Apr 2022;31(4):951-973. doi:10.1007/s11136-021-02926-x
15. Zimpel SA, Torloni MR, Porfírio GJ, Flumignan RL, da Silva EM. Complementary and alternative therapies for post-caesarean pain. *Cochrane Database Syst Rev*. Sep 1 2020;9(9):Cd011216. doi:10.1002/14651858.CD011216.pub2
16. Chou R, Wagner J, Ahmed AY, et al. AHRQ Comparative Effectiveness Reviews. *Treatments for Acute Pain: A Systematic Review*. Agency for Healthcare Research and Quality (US); 2020.
17. Smith CA, Hill E, Denejkina A, Thornton C, Dahlen HG. The effectiveness and safety of complementary health approaches to managing postpartum pain: A systematic review and meta-analysis. *Integr Med Res*. Mar 2022;11(1):100758. doi:10.1016/j.imr.2021.100758

© 2024 Mak S et al. *JAMA Network Open.*
