# Supplementary material for: Use of Massage Therapy for Pain, 2018-2023: A Systematic Review
Source: JAMA Netw Open. 2024 Jul 15;7(7):e2422259. doi: 10.1001/jamanetworkopen.2024.22259 (PMC11250267; doi:10.1001/jamanetworkopen.2024.22259)
Supplement: Supplement 2. — Data Sharing Statement [file jamanetwopen-e2422259-s002.pdf]

Data Sharing Statement

Mak. Use of Massage Therapy for Pain, 2018-2023: A Systematic Review. *JAMA Netw Open*. Published online July 15, 2024. doi:10.1001/jamanetworkopen.2024.22259

Data
Data available: No
